# Ten simple rules to host an inclusive conference

**DOI:** 10.1371/journal.pcbi.1010164

**Published:** 2022-07-21

**Authors:** Rocío Joo, Andrea Sánchez-Tapia, Sara Mortara, Yanina Bellini Saibene, Heather Turner, Dorothea Hug Peter, Natalia Soledad Morandeira, Matt Bannert, Batool Almazrouq, Elizabeth Hare, Laura Ación, Juan Pablo Narváez-Gómez, Marcela Alfaro Córdoba, Federico Marini, Rita Giordano, Silvia Canelón, Anicet Ebou, Adithi R. Upadhya, Joselyn Chávez, Janani Ravi

**Affiliations:** 1 Global Fishing Watch, Washington, District of Columbia, United States of America; 2 Instituto de Pesquisas Jardim Botânico do Rio de Janeiro, Rio de Janeiro, Brazil; 3 International Institute for Sustainability, Rio de Janeiro, Brazil; 4 rOpenSci, Berkeley, CA, United States of America; Universidad Nacional Guillermo Brown, Buenos Aires, Argentina; Instituto Nacional de Tecnología Agropecuaria, Anguil, La Pampa, Argentina; R-Ladies, California, United States of America; 5 MetaDocencia, Ciudad Autónoma de Buenos Aires, Argentina; 6 Department of Statistics, University of Warwick, Coventry, United Kingdom; 7 The R Foundation for Statistical Computing, Vienna, Austria; 8 Mountain Hydrology and Mass Movements, Swiss Federal Institute for Forest, Snow and Landscape Research WSL, Birmensdorf, Switzerland; 9 Instituto de Investigación e Ingeniería Ambiental, Universidad Nacional de San Martín—Consejo Nacional de Investigaciones Científicas y Técnicas, General San Martín, Buenos Aires, Argentina; 10 KOF Swiss Economic Institute, ETH Zurich, Zurich, Switzerland; 11 Institute of Systems, Molecular and Integrative Biology, University of Liverpool, Liverpool, United Kingdom; 12 King Abdullah International Medical Research Center, Riyadh, Saudi Arabia; 13 Dog Genetics LLC, Astoria, New York, United States of America; 14 Instituto de Cálculo, Facultad de Ciencias Exactas y Naturales, Universidad de Buenos Aires—Consejo Nacional de Investigaciones Científicas y Técnicas, Ciudad Autónoma de Buenos Aires, Argentina; 15 Departamento de Botânica, Instituto de Biociências, Universidade de São Paulo, São Paulo, Brazil; 16 Department of Statistics, University of California, Santa Cruz, California, United States of America; 17 Institute of Medical Biostatistics, Epidemiology and Informatics (IMBEI), University Medical Center Mainz, Mainz, Germany; 18 Royal Society of Chemistry, Thomas Graham House (290), Science Park, Milton Road, Cambridge, United Kingdom; 19 Department of Biostatistics, Epidemiology & Informatics, University of Pennsylvania, Philadelphia, Pennsylvania, United States of America; 20 Bioinformatics Team, Département de Formation et de Recherche Agriculture et Ressources Animales, Institut National Polytechnique Félix Houphouët-Boigny, Yamoussoukro, Côte d’Ivoire; 21 ILK Labs, Bengaluru, Karnataka, India; 22 Departamento de Microbiología Molecular, Instituto de Biotecnología UNAM, Cuernavaca, Morelos, Mexico; 23 Departments of Pathobiology and Diagnostic Investigation, Microbiology and Molecular Genetics, Michigan State University, East Lansing, Michigan, United States of America

## Abstract

Conferences are spaces to meet and network within and across academic and technical fields, learn about new advances, and share our work. They can help define career paths and create long-lasting collaborations and opportunities. However, these opportunities are not equal for all. This article introduces 10 simple rules to host an inclusive conference based on the authors’ recent experience organizing the 2021 edition of the useR! statistical computing conference, which attracted a broad range of participants from academia, industry, government, and the nonprofit sector. Coming from different backgrounds, career stages, and even continents, we embraced the challenge of organizing a high-quality virtual conference in the context of the Coronavirus Disease 2019 (COVID-19) pandemic and making it a kind, inclusive, and accessible experience for as many people as possible. The rules result from our lessons learned before, during, and after the organization of the conference. They have been written mainly for potential organizers and selection committees of conferences and contain multiple practical tips to help a variety of events become more accessible and inclusive. We see this as a starting point for conversations and efforts towards building more inclusive conferences across the world. * Translated versions of the English abstract and the list of rules are available in 10 languages in S1 Text: Arabic, French, German, Italian, Japanese, Korean, Portuguese, Spanish, Tamil, and Thai.

## Introduction

Conferences are spaces to meet and reconnect with members from a specific community, learn about advances in the field, and share recent contributions. A good conference experience can make a difference in the professional development of the participants and create long-lasting collaborations and opportunities. However, opportunities for participating in conferences are not equally available for all. Many academic and tech conferences have been spaces that reproduce systemic inequalities, by failing to overcome the barriers for participation and giving more opportunities to the most privileged individuals (typically white people from high-income backgrounds, prestigious institutions, power native English speakers with no disabilities) [1–4]. Like many others, the authors of this article have experienced these inequalities in conferences, from different perspectives and levels of inclusion/exclusion and privilege. We had the opportunity to organize a virtual and global statistical computing conference, useR! 2021 [5], for users and developers of the R programming language [6]. Coming from different backgrounds, career stages, and even continents, we embraced the challenge of organizing a high-quality virtual conference in the context of the Coronavirus Disease 2019 (COVID-19) pandemic and making it a kind, inclusive, and accessible experience for as many people as possible.

**Fig 1:**
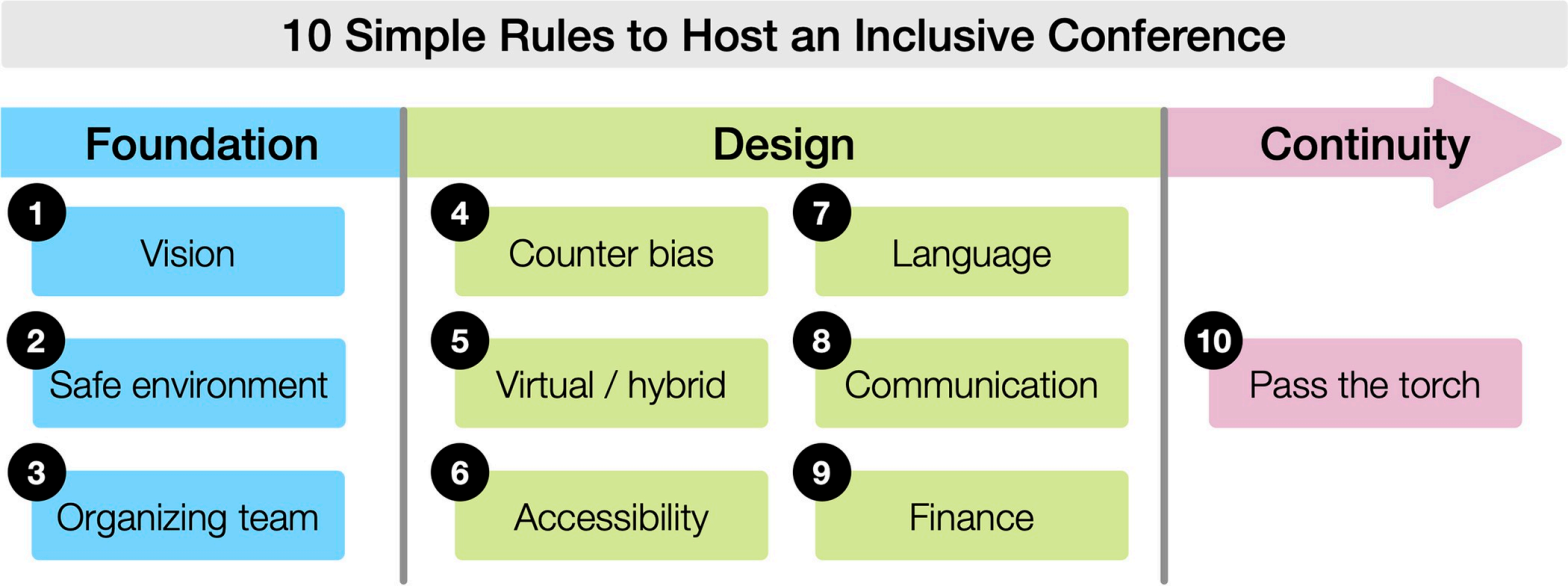
Schematic diagram of the rules organized in 3 groups: foundation (Rules 1 to 3), design (Rules 4 to 9), and continuity (Rule 10). See alt-text in the “Alternative text for figures” section.

Here, we present a set of 10 rules based on the lessons we learned before, during, and after the organization of the useR! conference. The rules were first drafted by the core team and the members of the diversity, accessibility, and inclusion team of useR! 2021 who wrote their own 10 simple rules (see S1 Text) based on the collective experience of organizing the conference and fueled by personal experiences and literature review. A final list was proposed and then discussed and reviewed by the rest of the coauthors. The rules are organized in 3 sections: 3 foundation rules, 6 design rules, and a continuity rule (**Fig 1**). The **foundation rules** comprise key elements to conceive the work on diversity and inclusion in any conference. **Rule 1** is about setting a vision of diversity and inclusion that should guide all the efforts and decision-making in the organization. **Rule 2** focuses on how to create a safe and welcoming environment for all the attendees. **Rule 3** highlights the importance of starting with an inclusive and diverse organizing team and provides tips on work dynamics. The **design rules** focus on weaving inclusion into the conference design process. In **Rule 4**, we introduce multiple ways to counteract bias in the conference program (keynotes, program committee, abstract selection, and thematic sessions). **Rule 5** provides advice for designing an inclusive online component in virtual and hybrid conferences. **Rule 6** focuses on accessibility practices to include people with disabilities. In **Rule 7**, we provide suggestions to account for linguistic diversity. **Rule 8** offers tips for developing an inclusive communication strategy. In **Rule 9**, we address budgeting for inclusive practices and helping participants with affordable registration costs, scholarships, and other forms of financial support. Finally, **Rule 10**, the **continuity** rule, emphasizes the importance of self-assessment and advocates for making the conference part of a long-term commitment to inclusion and for passing the torch to future organizers.

We believe that these rules can guide organizers who intend to put inclusion at the core of their conference right from the conception and planning phases. The rules will also help people who are part of meeting committees that oversee the site/location selection process, or that coordinate with the local organizers of conferences. We see the rules as a starting point for conversations and efforts towards building more inclusive conferences across the world.

## Rule 1: Set a vision for diversity and inclusion

Diversity encompasses multiple dimensions: age, disability, career stage, gender, gender identity, geographic origin, language, neurodiversity, race, religion, sexual orientation, and socioeconomic background, to name a few. Human diversity should be celebrated and respected in every way. Nonetheless, we live in a world with implicit hierarchies along these axes. Some statuses (e.g., being cisgender, white, male, from North America, or Western Europe) hold the privilege of being the defaults around which all systems—including conferences—are consciously and unconsciously built. People outside the dominant groups suffer different forms of oppression, like sexism, racism, ableism, homophobia, and transphobia, and sometimes these forms of oppression happen simultaneously and in complex ways (a concept known as “intersectionality” [7]). As a result, some groups of people have been systematically excluded from or only partially included in academic, scientific, and professional circles. Recognizing this systematic exclusion is paramount to inclusion.

Lack of diversity will vary across areas of knowledge and depend on the geographic context of the conference. Assess how it translates to the conference’s particular field and scientific or professional community. Some information can come from diversity, equity, and inclusion (DEI) studies in the field or at a larger scale (e.g., STEM—science, technology, engineering, and mathematics) if they exist. Most importantly, involve the community—the subset of the general public that would ideally attend the conference—to help make this assessment and set the vision and goals of diversity and inclusion for the conference. Define measurable outcomes for these goals (e.g., a gender distribution of your speakers that is representative of the general population, the participation of people from diverse races and ethnicities among the organizers, speakers, and attendees, or participation from key geographic regions). They will help you be accountable along the way (see **Rule 10**). The vision can be expressed in a diversity statement to be used as a reference for decision-making in all areas of the conference organization.

## Rule 2: Create a safe and welcoming environment

Real inclusion can only be achieved in an environment where everybody feels safe, respected, and invited to take part in the conference activities and community. Be proactive and design a welcoming conference experience that takes into account the well-being of all the attendees. This means respecting all the aspects included in your diversity vision (see **Rule 1**). For example, promote the use and respect of pronouns and commit to respect all genders and sexual orientations. If the conference has an in-person component, preassign quiet, private spaces for religious practices, lactation, and child care. Include menu options that account for diverse dietary and religious requirements. Some of these actions are very easy to implement and have a significant impact on making everyone feel truly included (see [8] for more examples and great advice on “low-hanging fruits”), some others need careful planning in advance, so it is good to have them in mind from the beginning. Open the schedule for events organized by thematic groups and communities (e.g., task force meetings, LGBTQIA+-friendly spaces, Black in STEM, women in science), and sessions aimed to welcome first-timers to the conference.

Creating a safe and welcoming environment also requires promoting a culture of support, including the idea that anyone can intervene respectfully in defense of other attendees in case anything unpleasant, inappropriate, or offensive happens. This is called “active bystandership,” consider providing training or preconference discussions to promote it. However, the most important step that you should take to ensure the safety of the community is adopting a code of conduct (CoC) and preparing a diverse response team to enforce it [9]. The CoC is a living document meant to keep the community safe and should state clearly the behaviors that are deemed unacceptable by the community, the consequences for engaging in such behaviors, and the way to report violations [10]. Bear in mind that every dimension of diversity can be a target of unacceptable behavior. The CoC should help honor the vision expressed in the diversity statement.

The CoC response team must receive training on how to receive reports, respond to incidents, and communicate their responses to the whole community. The team should be prepared early during conference planning because CoC violations can happen before the conference begins (e.g., on social media or in organization meetings). Responding to CoC reports can be one of the most emotionally intense tasks of the team. Resources permitting, consider adding funds for their work to your budget (see **Rule 9**). We strongly recommend reading “How to Respond to Code of Conduct Reports” [10] as an excellent starting point for building a strong CoC response team.

## Rule 3: Gather an inclusive and diverse organizing team

A genuinely inclusive conference can only be organized by an inclusive and diverse team. Organizers should comprise a team with people from as many different backgrounds as possible in terms of regions, genders, ethnicities, career stages, and other aspects of diversity. People with disabilities often say: “Nothing about us without us” [11,12]; the same holds for other dimensions of diversity. Having people from diverse backgrounds in decision-making roles will positively affect the conference as a whole because all the processes will benefit from their expertise, experience, and distinct perspectives [13]. The experience of people in marginalized groups is especially important; they cannot be replaced by good intentions or second-hand knowledge from people who have not lived through the same experiences [14]. In addition, a diverse team plays an essential role in creating a welcoming space because representation—seeing people with similar life experiences occupying critical positions of power and breaking negative stereotypes—is one of the best ways to create a sense of belonging for everyone participating in the conference. If you have already started assembling an organizing team, check for gaps in its composition and do your best to fill them. The regional and local communities, groups, or associations in your field are good sources to tap into.

Create sub-teams to distribute fairly the large amount of work that organizing a conference represents. Having a dedicated DEI team may be an excellent way to give the topic the attention and energy it requires. The core team should support the DEI initiatives and advocate for inclusion across all conference areas. It is important, though, to ensure that organizers with diverse backgrounds are not restricted to only work on diversity and inclusion aspects; every person should have the freedom to choose which areas of the conference they want to contribute to. When it comes to team leadership, do not expect self-nomination and voting to work as infallible mechanisms to fill these positions equitably. Instead, nominate folks directly and offer them leadership positions in the organizing team, especially those positions that would be typically occupied by people from privileged groups. Moreover, some people may lack the institutional or financial support to put time and effort into the organization tasks and do not have the luxury to commit for free; consider reallocating resources to give them a stipend for their work (see **Rule 9**). In any case, try to find meaningful ways to acknowledge everybody’s work. Use the conference spaces (e.g., webpage, opening/closing ceremony at the conference, social media) to give proper credit to the people who are making the event possible. And most importantly, take care of your team and their well-being! Check on them regularly and make sure that everyone is comfortable. This represents a great amount of work and you will need to support each other for the long haul.

## Rule 4: Consciously counteract bias in the conference program

The conference program may lack diversity in the invited roles—such as keynote speakers, program/scientific committee, session chairs, panelists—the accepted abstracts, and even the scope of the topics covered. It is important to counteract biases in all of these aspects. When choosing or inviting people for visible and valued roles in the conference, it is likely that there will not be much diversity in the first pool of names [15–18]. Rather than deterring you, this should encourage you to go beyond your current networks to look for, reach out to, invite, encourage, and onboard great people that are not routinely in the spotlight, making sure that these roles do not reinforce existing privileges.

Biases in the final list of abstracts begin with self-selection: Some potential conference participants may not feel confident enough to submit their work, especially to large and prestigious conferences. To mitigate such self-selection, some communities have pre-submission mechanisms to aid their members prepare and receive feedback for their abstract (e.g., the Latin-R and R-Ladies communities have volunteers to help their members improve their conference abstracts). If this does not exist in your field yet, consider asking members of your community to provide a similar kind of mentorship. To mitigate self-selection from non-native speakers, consider accepting submissions in several languages (see **Rule 7**).

The abstract review process can be subjected to implicit bias [19] when folks in the selection committee unconsciously assign a positive or negative value to the names and affiliations of authors—and their perceived origin, ethnicity, gender, or native language—affecting the chances to get their work accepted. Selection committees need to be reminded of such biases in the evaluation process, to try to avoid judging more scrupulously the work of people perceived as part of minoritized groups or being kinder when reviewing the abstracts from those perceived as part of privileged groups and considering their work as more relevant [16,20]. Evaluate the plausibility of anonymizing the submission information as much as possible and define the most appropriate strategy for reviewing (e.g., double-blind, double-open, or single-blind reviews; see [8]). Even well-meaning and carefully crafted processes can be prone to reproduce the biases present in academia and society. It is, therefore, essential to examine the final list of selected abstracts to check if there is a lack of diversity. If proposals with similar quality were still judged differently because of unconscious bias, consider giving preference to the work from people in minoritized groups. This manual examination at the final stage is unlikely to be reproducible. Nonetheless, the whole process should be as transparent as possible: Evaluation criteria should be clearly stated and shared with the reviewers and authors, preferably during the call for abstracts. It is worth noting that self-selection and unconscious bias may be aggravated by the use of video abstracts as an alternative to written ones, affecting people without the resources to create good quality videos, people with disabilities, non-native speakers, racialized people, and people with diverse body types, among others [21].

Bias can also be expressed in the level of visibility given to the diverse topics in the conference program. In many academic or technological fields, typically competitive activities or areas—such as scientific publications or software development—are more valued than those focused on sharing and cooperation, like community building, teaching, or mentoring. The latter activities are equally, if not more, relevant and challenging, and are usually performed by women, racialized people, people with disabilities, and other minoritized groups [22–24]. When planning the program for the conference, consider giving visibility to the whole range of activities and practitioners that contribute to the field by proposing new thematic sessions, broadening the scope of talks, keynotes, and tutorials.

## Rule 5: Design a strong online component

After experiencing virtual conferences mostly due to the COVID-19 pandemic, there have been multiple calls to retain an online component in conferences, either as completely virtual events or using hybrid formats (combining in-person and online components) [25–30]. Virtual conferences remove barriers for inclusion like costs of participation, the logistics of long-distance and international travel, and discriminatory visa applications [25,27,31,32]. Make sure that you are not creating additional barriers when choosing conference platforms, schedule, and activities. Check every platform’s screen reader accessibility (see **Rule 6**), technological requirements (a high-speed internet or a latest generation computer prerequisite could exclude attendees), and geopolitical restrictions depending on the scale of the conference (some services are not available in all countries). Be mindful of the different time zones and time constraints of the potential attendees and create flexible schedules. Avoid creating a strictly synchronous live program; ask for prerecorded talks, record sessions, and make them available during the conference.

If you are organizing a hybrid event, decide on the relative importance that online and in-person components will take [33]. The most inclusive practice would be to give equal weight to both components, articulating them well and maximizing the experience of everyone attending the conference. The audience, chairs, and presenters should all be able to interact regardless of their in-person or remote status. To best integrate in-person and remote attendees, all questions and comments should pass through a single online system. The physical venue would have to include multiple ways to connect with online folks such as cameras and microphones to allow them to follow who is speaking at the in-person space and tablets to help in-person attendees without suitable personal devices to interact with remote participants during Q&A or networking sessions.

Online networking and socializing can make people from minoritized groups feel more included, thus participate and contribute more, than when meeting face-to-face for the first time (e.g., [34,35]). Take advantage of the online component to have a broader range of social activities that can appeal to people with different backgrounds and preferences. Offer some activities that require voice or video interactions, and others that involve only written chat. You can adapt in-person activities for online and hybrid settings or create new activities. Virtual art exhibitions, yoga sessions, movie watch parties, trivia quizzes, and virtual city tours are just some examples. When planning for networking activities in hybrid events, try to promote connections between both groups of participants. It is understandable that a few of these activities would only be in-person, but there should be a fair proportion of activities connecting in-person and online attendees.

## Rule 6: Make the conference accessible to people with disabilities

Conferences are among the least accessible spaces that people with disabilities may encounter in professional contexts [36]. Even when conferences implement other inclusive practices, the participation of people with disabilities is often overlooked [37]. Including people with disabilities in the organizing team from the start can have a large impact (see **Rule 3**). Planning for accessibility requires time, experience, and early decision-making, because inaccessible features are extremely difficult to correct at later stages [4]. To ensure that disabled organizers can contribute substantially, the tools selected for behind-the-scenes organizing, communication, and planning the conference must be compatible with adaptive technology like screen readers. When working with deaf and hard-of-hearing people, spoken conversations should have captions.

If the conference has an in-person component, the venue should comply with common accessibility standards, such as being adequate for people who use wheelchairs, having signs in Braille, and using a sound system compatible with hearing devices and language interpretation, to name a few. Invisible disabilities (e.g., dyslexia, anxiety, attention-deficit/hyperactivity disorder (ADHD), autism) should be accounted for proactively, for example, by providing quiet spaces for privacy and noise-free conversations, or providing chairs in open spaces (see [38] for other examples).

Regardless of the conference format, all platforms (website, registration, abstract submission, chat system, conference administration tools, live streaming) should be screen reader-friendly and keyboard accessible. All images used in digital conference spaces should have alternative text and use colorblind-safe palettes. If there is video streaming, it should have good-quality (not automated) captions and a transcript; live presentations should have good-quality live captions too. If the conference has a regional scope, sign language interpreters can be a better option than captions.

Encourage the preparation of accessible slides and presentations by providing accessibility guidelines and presentation templates and be available for any questions that presenters and attendees may have; see [39] for an example of accessibility guidelines and [40–42] for further accessibility recommendations. Social events and networking should also have accessibility features like captioning or sign language interpretation, and include activities that do not restrict participation based on body type or ability.

Importantly, accessibility practices are inclusive not only for people with disabilities but also for everyone. For example, captions are helpful for non-native speakers and having slides available for download helps attendees with low bandwidth connection.

## Rule 7: Make room for the linguistic diversity of your community

In academic and technical events, the linguistic diversity of the participants is often overlooked. English is usually the official and sole language for submissions, presentations, tutorials, workshops, conference platforms, websites, and communications. While English is indeed regarded as the primary language in scientific communication and one official language makes it conducive to communicate widely, this makes being a native English speaker a privilege [43]. Non-native English speakers may miss opportunities to attend or to actively participate in conferences (e.g., presenting, asking questions, or taking part in discussions), and conferences may in turn miss innovative contributions.

Create an inclusive environment by encouraging the full participation of non-native English speakers. This may be done in multiple ways. Allow for abstract submission in both English and the applicant’s preferred native language, and whenever possible, assign a reviewer who is fluent in that language. Alternatively, the organizing team could define a set of accepted languages for abstract submission, and allow applicants to choose from this list. The goal should be to judge the abstracts primarily by the quality or relevance of the work instead of English proficiency. Whenever possible, identify other prominent languages for the conference and provide translated captions or live language interpretation into these key languages, including multilingual Q&A sessions. Another way to engage non-native English participants is to host sessions and events in languages other than English. Promote these sessions among all conference attendees and announce if captions will be available; non-English sessions should be given the same importance as the rest of the program. Importantly, consideration and respect of accents or linguistic mistakes can make a significant difference in the conference experience of non-native English speakers. Kindly remind your native English-speaking audience to be mindful of that.

This rule also applies to cases when English is not the official language of the conference (e.g., a Latin American conference with Spanish as the official or most popular language). No language should be a barrier for inclusion.

## Rule 8: Build an inclusive communication strategy

The communication strategy of your conference should aim to reach broader audiences and express the commitment to diversity and inclusion and the welcoming spirit of the conference. Actively reach out and promote the conference to communities that have been systematically excluded. Promote the abstract submission call beyond the usual communication channels and reach out to local groups and communities of practice to encourage submissions by their members. The members of your team should be able to decide which languages to emphasize and which social media platforms to utilize for the promotion of the conference (e.g., Twitter, Facebook, LinkedIn, conference website, mailing lists). During the conference, pay special attention to promoting the sessions led, chaired, and presented by people in minoritized groups, multilingual sessions, and the diversified thematic sessions (see **Rule 4**) to give them the same visibility as all other sessions.

Publish the diversity statement, code of conduct, accessibility guidelines, and options for financial support (see **Rule 9**). These guidelines will not only communicate the conference values and practices but also will act as a built-in mechanism to hold the organizers accountable. Be transparent with potential attendees, communicate the limitations of the conference to let them know what to expect, and outline ways in which the organizing team will try to mitigate these issues. For example, inform participants if the conference platform is not completely screen reader-friendly and let people know if you are offering help to navigate it, or highlight on the conference website if captions will be available for some talks but not all. Provide a point of contact to help clarify any questions regarding accessibility, financial support, and other issues affecting participation.

Use inclusive language—language free from words, phrases, or tones that reflect prejudiced or discriminatory views of particular people or groups—in all communications [44]. Become familiar with the terminology used for disabilities, racialized groups, gender and sexual orientations, terms that are preferred by each group, and the terms that should be avoided. Do not expect minoritized people to teach you and accept feedback without being offended. Inclusive language also encompasses avoidance of excessive and over-specific technical jargon and acronyms.

**Fig 2:**
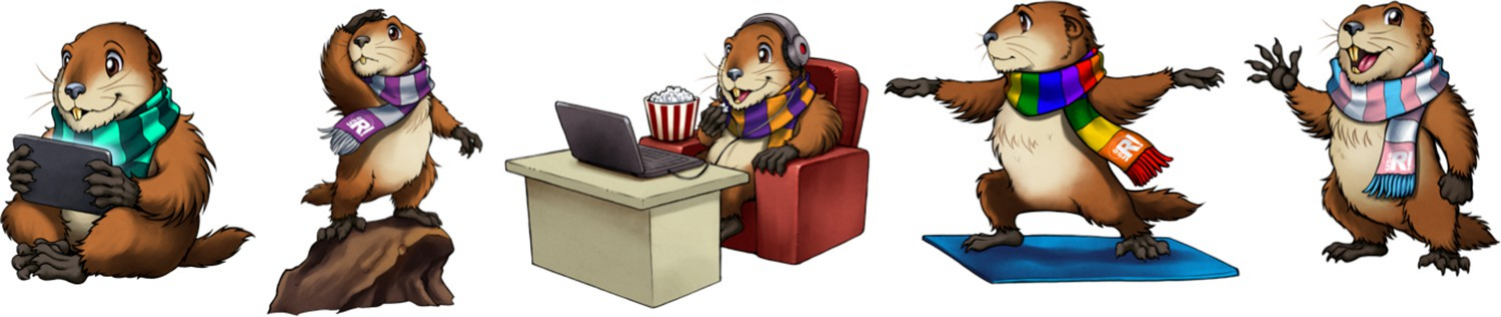
Margot the marmot was the useR! 2021 mascot. To represent the global and inclusive nature of the conference, Margot was drawn using scarves representing the colors of diverse communities of practice that are part of the large R community and specific groups of people we wanted to support explicitly. From left to right: Margot wearing scarves with the colors of MiR (https://mircommunity.com), R-Ladies (https://rladies.org), AfricaR (https://africa-r.org), and the LGBTQIA+ and transgender communities. Margot was created by Francisco Etchart and is available under a CC-BY-NC-SA license (https://github.com/useRconf/visuals-2021). See alt-text in the “Alternative text for figures” section. MiR, Minorities in R.

Communication should be fun! Try to develop creative ways to show that everyone is seen, respected, and welcome. For example, useR! 2021 created a mascot for the conference wearing different scarves reflecting folks from communities and various minoritized groups that were part of the potential attendees (**Fig 2**).

## Rule 9: Allocate adequate financial resources to support inclusion goals

Allocation of resources to support the diversity and inclusion vision and goals has to be intentional and demonstrate true commitment. Estimate the costs of these practices and define your priorities in advance (e.g., paying the organizing team, code of conduct training, captioning, accessible software, scholarships). An online conference might reduce overall costs (e.g., no rental costs for a physical venue), allowing to redirect the money towards inclusive practices. When asking for sponsorship, it might be easier to justify supporting concrete actions towards inclusion than making generic requests for funding.

When determining the registration rates, the socioeconomic context of participants, their career status, and their country of origin—if applicable—should be taken into account [30,45,46] (see [47] for an example of conversion rates based on country of origin and career status). Resources permitting, enable a “pay what you can” option. You could also aim to have a conference with no registration costs; this could especially apply for online events, but bear in mind that free events have a lower attendance rate than paid events [48]. Scholarships to attend the in-person component of the conference are an additional way to boost participation of people from minoritized groups by offering support for travel and lodging expenses. Design the diversity-related scholarship to be explicit about the groups you want to support and be transparent about the criteria for evaluation to avoid self-selection (refraining from applying). To assign these scholarships, conferences usually ask for cover letters or applications, which can be time-consuming and emotionally demanding. Simplifying the process of requesting financial support, especially for people who lack the time and resources (e.g., because of family responsibilities) could greatly increase their chances of applying for the support. Additional aid for attendees could also be offered—e.g., paying for child care or internet connection services for the online component. Bear in mind that transferring money internationally could be a cumbersome administrative process and significantly augment the burden of already marginalized groups. Whenever possible, facilitate alternate ways for money transfer (e.g., book flights, hotel reservations, waive conference fees, give gift cards).

## Rule 10: Make the conference part of a long-term process for inclusion

As conference organizers, one of the key post-conference tasks is to make a final assessment of the achievement of the diversity and inclusion goals. You can rely on the diversity goals defined at the beginning of the planning process (**Rule 1**). You will likely be able to evaluate the outcomes associated with these goals directly from the information collected from the organizing team, speakers, and participants (e.g., city/state/country of origin or preferred language). You may have set goals concerning how welcomed and included participants felt during the conference, and need to assess if the implemented practices translated into true inclusion. Surveys, focus groups, and informal conversations during and after the conference are good ways to collect this information and great opportunities to receive feedback from the attendees.

The feedback received from the participants and the whole assessment of the conference should be documented and shared with those who helped define the inclusion goals, the attendees, and future organizers. Ensure that the participants’ privacy and anonymity are respected; ask for consent and consider binning/grouping the data into categories when there is a low number of respondents. Gather and do your best to organize all the documentation produced during the organization process—or even after. Timelines, checklists, budgeting tips, email templates, accessibility checks, and guidelines, are a few of many resources that can be shared with your conference successors. Create a list with contact information of the members of the organizing team, as well as previous and potential speakers—if they agree to share their contact information—and pass it along. For conference series, it could be a good idea to create an official document (e.g., a wiki, knowledge base, or similar guide; see, for example, [5]) that can be used and kept up to date by future organizers. If you are part of a stable meeting committee (overseeing multiple editions), encourage organizers to follow these Rules and set up new standards for inclusion: Be explicit about the inclusive spirit of the conference and the practices that future organizers should commit to when publishing the call for conference organizers and defining the selection criteria.

## Concluding remarks

This article recommends changes in conference planning to support greater diversity and inclusion. Organizing a conference and implementing inclusive practices are both learning experiences. As conference organizers ourselves, we started with different levels of clarity about the principles and practices described here, learned many of them together during the organization phase, and learned even more when translating them into 10 rules for this paper. We hope that they can be useful guidelines to improve diversity and inclusion in your conference, and that you can adapt them, improve them, and share your lessons and experience with as many people as possible.

Be aware of all the factors that may hinder your efforts for inclusion: systemic discrimination, time or money constraints, geopolitical or public health contexts, or personal issues affecting your organizing team. Try to account for these factors as best as you can to set bold yet realistic goals of diversity and inclusion for the conference. Most importantly, your efforts will enhance the conference experience for all your attendees. They will notice the welcoming and inclusive spirit of the conference. Remember that inclusive conferences can have lasting impacts in career paths and create a sense of belonging in the community. That is more than enough motivation to make the effort worth it.

## Supporting information

S1 Text Abstract and list of rules in multiple languages and rules proposed in the process of shaping these 10 simple rules. (PDF)

## Alternative text for the figures

**Fig 1.** Diagram of the 10 rules organized into 3 groups: foundation rules (Rules 1 to 3), design rules (Rules 4 to 9), and a continuity rule (Rule 10). The overall diagram depicts an arrow from left to right containing all the rules. In the left column, under the first label “Foundation,” there are 3 text boxes in light blue: “1. Vision,” “2. Safe environment,” and “3. Organizing Team.” In the middle column, under the label “Design Rules” there are 6 light green boxes with Rules “4. Counter bias,” “5. Virtual/hybrid,” “6. Accessibility,” “7. Language,” “8. Communication,” “9. Finance.” To the right, below the arrowhead labeled “Continuity,” is the 10th Rule in a light pink box “10. Pass the torch.”

**Fig 2.** Five “Margot the marmot” with colorful scarves. From left to right: a marmot with a dark and light green scarf of the MiR community is looking at her tablet, a marmot with the purple and gray scarf of R-Ladies is stepping on a rock and watches the horizon, a marmot with an orange and purple AfricaR scarf is sitting on a couch watching a film on her laptop with popcorn, a yogi marmot with a rainbow LGBTQIA+ scarf does the warrior pose on her blue mat, and finally, a marmot with a scarf with the pink, light blue, and white color of the transgender pride flag waves her hand.
