# Supplementary material for: Ten simple rules to host an inclusive conference
Source: PLoS Comput Biol. 2022 Jul 21;18(7):e1010164. doi: 10.1371/journal.pcbi.1010164 (PMC9302732; doi:10.1371/journal.pcbi.1010164)
Supplement: S1 Text — (PDF) [file pcbi.1010164.s001.pdf]

Ten simple rules to host an inclusive conference

Rocío Joo[#,*], Andrea Sánchez-Tapia[#], Sara Mortara, Yanina Bellini Saibene, Heather Turner, Dorothea Hug Peter, Natalia Soledad Morandeira, Matthias Bannert, Batool Almazrouq, Elizabeth Hare, Laura Ación, Juan Pablo Narváez-Gómez, Marcela Alfaro Córdoba, Federico Marini, Rita Giordano, Silvia Canelón, Anicet Ebou, Adithi Upadhya, Joselyn Chávez, Janani Ravi*

[#]Co-primary authors with equal contribution.

*Co-corresponding authors: rocio.joo@globalfishingwatch.org, janani@msu.edu

Supporting Information | Table of Contents

1. ABSTRACT AND LIST OF RULES IN MULTIPLE LANGUAGES **2**

ENGLISH VERSION 2
اللغة العربية [ARABIC VERSION] 3
VERSION FRANÇAISE [FRENCH VERSION] 4
DEUTSCHE VERSION [GERMAN VERSION] 5
VERSIONE ITALIANA [ITALIAN VERSION] 6
日本語版 [JAPANESE VERSION] 7
한국어 버전 [KOREAN VERSION] 9
VERSÃO EM PORTUGUÊS [PORTUGUESE VERSION] 11
VERSIÓN EN ESPAÑOL / CASTELLANO [SPANISH VERSION] 12
தமிழ் மொழி பெயர்ப்பு [TAMIL VERSION] 13
ฉบับภาษาไทย [THAI VERSION] 15

2. RULES PROPOSED IN THE PROCESS OF SHAPING THESE TEN SIMPLE RULES **17**

PERSON 1 17
PERSON 2 17
PERSON 3 18
PERSON 4 18
PERSON 5 18
PERSON 6 19
PERSON 7 19
PERSON 8 20
PERSON 9 20
PERSON 10 21
PERSON 11 21

1. Abstract and list of rules in multiple languages

English version

Rocío Joo, Andrea Sánchez-Tapia, Sara Mortara, Yanina Bellini Saibene, Heather Turner, Dorothea Hug Peter, Natalia Soledad Morandeira, Matthias Bannert, Batool Almazrouq, Elizabeth Hare, Laura Ación, Juan Pablo Narváez-Gómez, Marcela Alfaro Córdoba, Federico Marini, Rita Giordano, Silvia Canelón, Anicet Ebou, Adithi Upadhya, Joselyn Chávez, Janani Ravi

Conferences are spaces to meet and network within and across academic and technical fields, to learn about new advances, and share our work. They can help define career paths and create long-lasting collaborations and opportunities. However, these opportunities are not equal for all. This article introduces ten simple rules to host an inclusive conference based on the authors' recent experience organizing the 2021 edition of the useR! statistical computing conference, which attracted a broad range of participants from academia, industry, government, and the non-profit sector. Coming from different backgrounds, career stages, and even continents, we embraced the challenge of organizing a high-quality virtual conference in the context of the COVID-19 pandemic and making it a kind, inclusive, and accessible experience for as many people as possible. The rules result from our lessons learned before, during, and after the organization of the conference. They have been mainly written for potential organizers and selection committees of conferences and contain multiple practical tips to help a variety of events become more accessible and inclusive. We see this as a starting point for conversations and efforts towards building more inclusive conferences across the world.

Rules

1. Set a vision for diversity and inclusion
2. Create a safe and welcoming environment
3. Have an inclusive and diverse organizing team
4. Consciously counteract bias in the conference program
5. Design a strong online component
6. Make the conference accessible to people with disabilities
7. Make room for the linguistic diversity of your community
8. Build an inclusive communication strategy
9. Allocate adequate financial resources to support inclusion goals
10. Make the conference part of a long-term process for inclusion

اللغة العربية [Arabic Version]

عشر قواعد سهلة لتأسيس مؤتمر شمولي ومتنوع

Batool Almarzouq

بتول محمد ال مرزوق

المؤتمرات العلمية هي مساحات مفتوحة للالتقاء والتواصل بين المهتمين في المجالات الأكاديمية والتقنية، للتعرف على التطورات الحديثة، ومشاركة أعمالهم مع المجتمع العلمي والتقني العالمي، هذه المؤتمرات تُساهِم في خلق مسارات وظيفية وفرص تعاون طويلة الأمد، لكنه -مع الأسف- في الكثير من الأحيان لا تكون هذه الفرص متساوية للجميع، هذه المقالة تقدم عشر قواعد بسيطة لتأسيس مؤتمر شامل ومتنوع، حيث استلهمنا هذه القواعد من تجربتنا نحن المنظمون لمؤتمرuseR! المهتم بالحوسبة الإحصائية في عام ٢٠٢١ والذي استقطب مجموعة واسعة من المشاركين من الأوساط الأكاديمية، والتقنية، والصناعية، والحكومية، والقطاع غير الربحي، وبرُغم أننا المنسقين للمؤتمر حضرنا من خلفيات متنوعة ومراحل علمية ومجالات مهنية مختلفة وأيضا مساحات جغرافية متباعدة ، لقد تبنينا التحدي المتمثل في تنظيم مؤتمر افتراضي عالي الجودة في سياق جائحة COVID-19 وجعلنا منه تجربة فريدة وشاملة وسهلة المنال لأكبر عدد ممكن من الأشخاص، هذه القواعد العشرة هي نتيجة وخلاصة للدروس التي تعلمناها قبل وفي خلال تنظيم المؤتمر وأيضا بعد ختامه، لقد تمت كتابة هذه القواعد بشكل أساسي للمنظمين أو لجان التنسيق حيث سيجدون فيها العديد من النصائح العملية والتي تُمكنهم من تنظيم أنواع مختلفة من الأنشطة وجعلها شمولية، وسهلة المنال لأكبر جمهور قدر الإمكان، ولعل هذه المقالة العلمية تكون نقطة انطلاق لجهود أكبر نحو بناء المزيد من المؤتمرات الشمولية في جميع أنحاء العالم.

القواعد العشرة

١- ضع رؤية للتنوع والشمول .
٢ - إخلق بيئة آمنة ومُرحِبة.
٣ - كوِّن فريق تنظيم شامل ومتنوع للمؤتمر.
٤ - تصدى بوعي لاي تحيز محتمل يتواجد في البرنامج التقديمي.
٥ - إدخال عنصر الأنترنت في جزء من المؤتمر.
٦- إجعل المؤتمر سهل المنال لذوي الإعاقة.
٧ - إفساح المجال للتنوع اللغوي في برنامج المؤتمر.
٨ - بناء استراتيجية اتصال شامله بين المشاركين.
٩ - تخصيص الموارد المالية الكافية لدعم أهداف البرنامج الشامل .
١٠- اجعل المؤتمر جزءًا من عملية طويلة المدى لتحقيق أهداف التنوع والشمولية.

Version Française [French version]

Dix règles simples pour organiser une conférence inclusive

Anicet Ebou, Rocío Joo, Andrea Sánchez-Tapia

Les conférences sont des espaces de rencontre et de mise en réseau au sein et entre les domaines académiques et techniques, pour s'informer des nouvelles avancées et partager des travaux. Elles peuvent aider à définir des parcours professionnels et créer des collaborations ainsi que des opportunités durables. Cependant, ces opportunités ne sont pas égales pour tout le monde. Cet article présente dix règles simples pour organiser une conférence inclusive. Nous avons récemment organisé l'édition 2021 de la conférence d'informatique statistique useR!, attirant un large éventail de participants provenants du monde universitaire, de l'industrie, du gouvernement et du secteur non lucratif. Issus de différents milieux, de différents niveaux de carrière et même de différents continents, nous avons relevé le défi d'organiser une conférence virtuelle de haute qualité dans le contexte de la pandémie de COVID-19 et d'en faire une expérience agréable, inclusive et accessible au plus grand nombre. Ces règles sont le fruit des enseignements que nous avons tirés avant, pendant et après l'organisation de la conférence. Elles ont été principalement rédigées à l'intention des potentielles équipes organisatrices ou comités de sélection de conférences et contiennent de nombreux conseils pratiques pour aider divers types d'événements à devenir plus accessibles et inclusifs. Nous considérons que ces règles constituent un point de départ pour des conversations et des efforts visant à construire des conférences plus inclusives à travers le monde.

Règles

1. Définir une vision de la diversité et de l'inclusion.
2. Créer un environnement sûr et accueillant.
3. Avoir une équipe d'organisation inclusive et diversifiée.
4. Contrer consciemment les préjugés dans le programme de la conférence.
5. Concevoir une forte composante en ligne.
6. Rendre la conférence accessible aux personnes handicapées.
7. Faire place à la diversité linguistique de votre communauté.
8. Élaborer une stratégie de communication inclusive.
9. Allouer des ressources financières suffisantes pour soutenir les objectifs d'inclusion.
10. Inscrire la conférence dans un processus d'inclusion à long terme.

Deutsche Version [German version]

Zehn einfache Regeln für die Organisation einer inklusiven Konferenz

Dorothea Hug Peter, Matthias Bannert

Konferenzen sind Orte, an denen wir uns in unseren akademischen und technischen Fachbereichen treffen und vernetzen, uns über neue Erkenntnisse informieren und unsere Arbeit teilen können. Sie können dazu beitragen, Karrierewege zu definieren und dauerhafte Kooperationen und Möglichkeiten zu schaffen. Diese Möglichkeiten sind jedoch nicht für alle gleich. In diesem Artikel werden zehn einfache Regeln für die Ausrichtung einer integrativen Konferenz vorgestellt. Die Autoren haben kürzlich die Ausgabe 2021 der useR! Konferenz für Statistical Computing organisiert, die ein breites Spektrum von Teilnehmern aus dem akademischen Bereich, der Industrie, der Verwaltung und dem gemeinnützigen Sektor angesprochen hat. Von unterschiedlichen Hintergründen, Karrierestufen und sogar Kontinenten aus haben wir uns der Herausforderung gestellt, eine hochwertige, virtuelle Konferenz im Kontext der COVID-19-Pandemie zu organisieren und unsere Veranstaltung zu einer freundlichen, inklusiven und zugänglichen Erfahrung für so viele Menschen wie möglich zu machen. Die Regeln resultieren aus den Erfahrungen, die wir vor, während und nach der Organisation der Konferenz gemacht haben. Sie wurden hauptsächlich für potenzielle Organisatoren oder Auswahlkomitees von Konferenzen verfasst und enthalten zahlreiche praktische Tipps, die dazu beitragen können, dass verschiedene Arten von Veranstaltungen so zugänglich und integrativ werden, wie sie sein können und sollten.

Regeln

1. Eine Vision für Diversität und Inklusion festlegen
2. Eine sichere und einladende Umgebung schaffen
3. Ein inklusives und vielfältiges Organisationsteam zusammenstellen
4. Vorurteilen im Konferenzprogramm bewusst entgegenwirken
5. Eine starke Online-Komponente entwickeln
6. Die Konferenz für Menschen mit Behinderungen zugänglich machen
7. Raum für die sprachliche Vielfalt der Teilnehmer*innen schaffen
8. Eine integrative Kommunikationsstrategie entwickeln
9. Angemessene finanzielle Mittel zur Unterstützung der Inklusionsziele bereitstellen
10. Die Konferenz zu einem Teil eines langfristigen Prozesses hin zu mehr Inklusion machen.

Versione italiana [Italian version]

Dieci semplici regole per organizzare una conferenza inclusiva

Federico Marini, Rita Giordano

Le conferenze sono spazi per incontrarsi e creare dei contatti sociali all'interno e attraverso ambiti accademici e tecnici, per conoscere nuovi progressi e sviluppi, e far conoscere e condividere il nostro lavoro. Le conferenze possono aiutare a definire al meglio i percorsi di carriera, creando collaborazioni e opportunità durature. Tuttavia, queste opportunità non sono uguali per tutti. Questo articolo introduce dieci semplici regole per organizzare una conferenza inclusiva. Gli autori hanno recentemente collaborato per organizzare l'edizione 2021 della conferenza useR!, incentrata sul linguaggio R per l'analisi statistics dei dati, attirando un'ampia gamma di partecipanti dal mondo accademico, industriale, governativo e del settore non profit. Gli organizzatori di useR!, provenienti da contesti, fasi di carriera e persino continenti diversi, hanno accettato la sfida di organizzare una conferenza virtuale di alta qualità nel contesto creato dalla pandemia di COVID-19, con l'obiettivo di renderla un'esperienza ospitale, inclusiva e accessibile per quante più persone possibile. Queste regole risultano dalle lezioni apprese prima, durante e dopo l'organizzazione della conferenza, e sono state stilate principalmente per potenziali organizzatori o comitati di selezione di conferenze, contenendo suggerimenti pratici per facilitare che altri eventi di natura simile possano diventare quanto più accessibili e inclusivi, così come d'altronde dovrebbero essere concepiti.

Le dieci semplici regole

1. Stabilire una visione riguardo la diversità e l'inclusione
2. Creare un ambiente sicuro e accogliente
3. Avere un team organizzativo che sia inclusivo e diversificato
4. Contrastare consapevolmente i pregiudizi nella creazione del programma della conferenza
5. Progettare una forte componente online
6. Rendere la conferenza accessibile alle persone con disabilità
7. Fare spazio alla diversità linguistica della tua comunità
8. Costruire una strategia di comunicazione inclusiva
9. Allocare risorse finanziarie adeguate per supportare gli obiettivi di inclusione
10. Rendere la conferenza parte di un processo a lungo termine per garantire l'inclusione

日本語版 [Japanese version]

いろいろな人が参加できるカンファレンスを主催するための10のシンプルなルール

Koki Tsuyuzaki, Kozo Nishida

カンファレンスは、学術・技術の分野内および分野間で出会い、ネットワークを構築し、新しい進歩について学び、作品を共有するための場です。それは、キャリアパスの意味を明確にし、長続きするコラボレーションや良い機会を作るのに役立ちます。 しかしながら、このような機会は誰にでも平等にあるわけではありません。この記事では、いろいろな人が参加できるカンファレンスを主催するための10のシンプルなルールを紹介します。そのルールは最近の筆者らの2021年度 useR! という統計学計算のカンファレンスの開催経験に基づいています。その useR! は学術界、産業界、政府、非営利セクターから幅広い参加者を集めました。 useR! にはさまざまなバックグラウンド、キャリアステージ、さらには大陸からの参加があり、COVID-19というパンデミックの状況の中でハイクオリティなバーチャルカンファレンスを開催し、できるだけ多くの人々にとって親切でいろいろな人が参加できるアクセシブルなものにするという課題に私達は取り組みました。このルールは、会議の開催前、開催中、そして開催後に私達が学んだ教訓から生まれたものです。このルールは、将来カンファレンスの主催者や選考委員会になる見込みのある人のために主に書かれており、様々なイベントが、よりアクセシブルでいろいろな人が参加できるようになるのを助けるための、複数の実用的なヒントを含んでいます。私達はこれを、世界中でよりいろいろな人が参加できるカンファレンスを作り上げていくための話し合いと取り組みの出発点であると考えています。

ルール

1. 多様性とその受け入れのためのビジョンを設定する
2. 安全で居心地の良い環境を作る
3. いろいろな人が参加できて多様な企画チームを持つ
4. カンファレンスプログラムにおけるバイアスを意識して防ぐ
5. 強いオンライン要素をデザインする

6. 障がいを持つ人々にもアクセシブルなカンファレンスにする
7. コミュニティの言語的多様性に配慮する余地を残しておく
8. いろいろな人が参加できるコミュニケーション戦略を立てる
9. 多様性を受け入れる目標を支援するために十分な財源を割り当てる
10. カンファレンスを多様性を受け入れるための長期的なプロセスの一部とする

한국어 버전 [Korean version]

포용적인 컨퍼런스를 개최하는 10가지 간단한 규칙

KwangChun Lee

컨퍼런스는 학문과 기술 분야를 넘나들며 만나고 네트워킹하며 새로운 진보된 기술을 배우고 서로의 연구성과물을 공유하는 공간이다. 컨퍼런스를 통해서 직업경력을 정의내리기도 하고 지속가능한 협업은 물론 새로운 기회를 만들 수 있다. 하지만, 이런 기회가 모두에게 공평하지는 않다. 포용적인 컨퍼런스를 개최하는 10가지 간단한 규칙을 소개한다. 최근 저자는 2021년 useR! 통계 컴퓨팅 컨퍼런스를 조직하면서 학계, 산업계, 정부, 비영리 분야로부터 폭넓은 참여자를 이끌어내는 역할을 담당했다. 경력단계, 대륙, 배경이 서로 다른 상황과 코로나19라는 특수성이 있는 상황에서 고품질 비대면 컨퍼런스를 체계적으로 조직하고 가능하면 많은 참여자들에게 접근성, 포용성, 인간적인 면까지 고려하는 도전적인 과제를 수행했다. 다음 규칙은 컨퍼런스를 조직하는 과정은 물론 전과 후에 배운 내용에 기초한다. 잠재적인 컨퍼런스 주최측 혹은 컨퍼런스 심의위를 주된 대상으로 삼고 있으며 다수 실무적인 조언도 포함되어 있다. 이를 통해서 범세계적으로 좀더 포용적인 컨퍼런스를 만들어나가는데 필요한 노력과 논의점에 대한 출발점으로 삼아주셨으면 좋겠다.

규칙

1. 다양성과 포용성에 대한 비전을 설정하라.
2. 안전하고 반갑게 맞이하는 환경을 구축하라.
3. 포용적이며 다양성을 갖는 조직위를 팀으로 구성하라.
4. 컨퍼런스 프로그램에 담긴 편향에 지각있게 대응하라.
5. 강력한 온라인 구성요소를 설계하라.
6. 장애를 갖는 분들도 접근할 수 있도록 컨퍼런스를 만들어라.
7. 커뮤니티에 언어적인 다양성을 담을 수 있는 공간을 만들어라.
8. 포용적인 커뮤니케이션 전략을 구축하라.

9. 포용목표를 지탱할 수 있도록 재정을 적절한 할당하라.
10. 컨퍼런스의 일부를 포용성을 넓힐 중장기 과정으로 만들어라.

Versão em Português [Portuguese version]

Dez regras simples para organizar uma conferência inclusiva

Sara Mortara, Andrea Sánchez-Tapia

Conferências são espaços para conhecer e se conectar com áreas acadêmicas e técnicas, aprender sobre novos desenvolvimentos e compartilhar nosso trabalho. Uma conferência pode definir carreiras e criar colaborações e oportunidades de longa duração. Entretanto, essas oportunidades não são iguais para todas as pessoas. Neste artigo, apresentamos dez regras simples para organizar uma conferência inclusiva. Nós recentemente organizamos a edição de 2021 da conferência de computação estatística useR!, que atraiu uma ampla gama de participantes da academia, indústria, setor público e organizações não-governamentais. Vindo de diferentes experiências, estágio de carreira e até continentes, nós aceitamos o desafio de organizar uma conferência virtual de alta qualidade no contexto da pandemia de COVID-19, criando uma experiência amigável, inclusiva e acessível para o maior número de pessoas possível. As regras resultam das lições que aprendemos antes, durante e depois da organização da conferência e foram escritas principalmente para pessoas envolvidas na organização e comitês de seleção de conferências. Elas contêm múltiplas dicas práticas que podem ajudar diversos tipos de eventos a se tornarem mais acessíveis e inclusivos. Consideramos que elas constituem um ponto de partida para conversas e esforços que ajudem a criar conferências mais inclusivas ao redor do mundo.

Regras

1. Defina uma visão de diversidade e inclusão
2. Crie um ambiente seguro e acolhedor
3. Reúna um comitê organizador inclusivo e diverso
4. Combata conscientemente os vieses no programa da conferência
5. Programe um componente on-line forte
6. Faça a conferência acessível para pessoas com deficiência
7. Crie espaço para diversidade linguística na sua comunidade
8. Construa uma estratégia de comunicação inclusiva
9. Reserve recursos financeiros para apoiar os objetivos de inclusão
10. Faça a conferência parte de um processo de inclusão de longo prazo

Versión en español / castellano [Spanish Version]

Diez reglas simples para organizar una conferencia inclusiva

Natalia Soledad Morandeira, Yanina Bellini Saibene

Las conferencias son espacios para el encuentro y para construir redes dentro y a través de campos académicos y técnicos, para aprender sobre nuevos avances y para compartir nuestro trabajo. Pueden ayudar a definir trayectorias profesionales y a crear oportunidades y colaboraciones duraderas. Sin embargo, estas oportunidades no son iguales para todas las personas. El presente artículo presenta diez reglas simples para organizar una conferencia inclusiva. Las/los autoras/es recientemente organizamos la edición 2021 de la conferencia useR! de computación y estadística, que atrae un amplio rango de participantes de la academia, la industria, organizaciones gubernamentales y el sector sin fines de lucro. Procedentes de diferentes ámbitos, etapas profesionales e incluso continentes, nos comprometimos con el desafío de organizar una conferencia virtual de alta calidad en el contexto de la pandemia por COVID-19, y hacer de ella una experiencia amable, inclusiva y accesible para la mayor cantidad de personas posible. Las reglas son el resultado de las lecciones aprendidas antes, durante y después de la organización de la conferencia. Fueron escritas para potenciales integrantes de equipos de organización o de comités de selección de conferencias. Contienen múltiples consejos prácticos que pueden ayudar a que diferentes tipos de eventos sean más accesibles e inclusivos. Consideramos que este es un punto de partida para las conversaciones y los esfuerzos encaminados a crear conferencias más inclusivas en todo el mundo.

Reglas

1. Definir una visión sobre diversidad e inclusión
2. Crear un ambiente seguro y acogedor
3. Tener un equipo de organización diverso e inclusivo
4. Contrarrestar conscientemente los sesgos en el programa de la conferencia
5. Diseñar un sólido componente virtual
6. Hacer que la conferencia sea accesible para personas con discapacidades
7. Generar espacios para la diversidad lingüística de tu comunidad
8. Construir una estrategia de comunicación inclusiva
9. Asignar recursos financieros adecuados para sostener los objetivos de inclusión
10. Hacer que la conferencia sea parte de un proceso de inclusión a largo plazo

தமிழ் மொழி பெயர்ப்பு [Tamil version]

உள்ளடக்கிய மாநாட்டை நடத்த பத்து எளிய விதிகள்

Arjun Krishnan / *அர்ஜுன் கிருஷ்ணன்*

மாநாடுகள் என்பது கல்வி மற்றும் தொழில்நுட்பத் துறைகளுக்குள்ளும், இடையேயும் சந்திப்பதற்கும், தொடர்புகள் உருவாக்குவதற்கும், புதிய முன்னேற்றங்களைப் பற்றி அறிந்து கொள்வதற்கும், தங்கள் பங்களிப்புகளை பகிர்ந்து கொள்வதற்குமான தளங்கள். தொழில் பாதைகளை வரையறுக்கவும், நீண்டகால ஒத்துழைப்பு மற்றும் வாய்ப்புகளை உருவாக்கவும் இத்தளங்கள் உதவும். இருப்பினும், இத்தகைய வாய்ப்புகள் அனைவருக்கும் சமமாகக் கிடைப்பதில்லை. 2021 ஆம் ஆண்டு யூஸ்ஆர் (useR!) புள்ளியியல் கணக்கியல் மாநாட்டை சமீபத்தில் ஏற்பாடு செய்ததன் அனுபவத்தையொட்டி எழுதப்பட்ட இந்தக் கட்டுரை, வாய்ப்புகளையும் வளங்களையும் சமமாக வழங்கும் நோக்கை உள்ளடக்கிய உள்ளடக்கிய மாநாட்டை நடத்துவதற்கான பத்து எளிய விதிகளை அறிமுகப்படுத்துகிறது. யூஸ்ஆர் மாநாடு கல்வித்துறை, தொழில்துறை, அரசு மற்றும் இலாப நோக்கற்றத் துறைகளிலிருந்து பரந்தளவிலான பங்கேற்பை ஈர்க்கிறது. வெவ்வேறு பின்னணிகள், தொழில் நிலைகள், மற்றும் கண்டங்களிலிருந்து வரும் நாங்கள், கோவிட்-19 தொற்றுநோயின் பின்னணியில் உயர்தர மெய்நிகர் மாநாட்டை ஏற்பாடு செய்வதன் சவாலை ஏற்றுக்கொண்டு, அதை ஒரு ஆதரவான, உள்ளடக்கிய, மற்றும் முடிந்தவரை பலரும் அணுகக்கூடிய அனுபவமாக அமைத்தோம். மாநாட்டை ஒழுங்கமைப்பதற்கு முன்பும், அதன்போதும், பின்பும் நாங்கள் கற்றுக்கொண்டப் பாடங்களிலிருந்து விளைந்தன இவ்விதிகள். இவை முக்கியமாக மாநாடுகளின் அமைப்பாளர்கள் மற்றும் தேர்வுக் குழுக்களுக்காக எழுதப்பட்டவை. பல நடைமுறைக் குறிப்புகளைக் கொண்டிருக்கும் இக்கட்டுரை பல்வேறு வகையான நிகழ்வுகளை மேலும் அணுகக்கூடியதாகவும் உள்ளடக்கியதாகவும் அமைக்க உதவும். உலகளவில் மெம்மேலும் உள்ளடக்கிய மாநாடுகள் அமைப்பதற்கான உரையாடல்களும் முயற்சிகளுக்கும் ஓர் ஆரம்பமாக நாங்கள் இதைப்பார்க்கிறோம்.

விதிகள்

1. பன்முகத்தன்மை மற்றும் உள்ளடக்கத்திற்கான தொலைநோக்குப் பார்வையை அமைக்கவும்
2. பாதுகாப்பான மற்றும் வரவேற்கத்தக்க சூழலை உருவாக்கவும்
3. உள்ளடக்கிய மற்றும் பன்முகப்பட்ட அமைப்பாளர் குழுவைக் கொண்டிருக்கவும்
4. மாநாட்டுத் திட்டத்தில் உள்ள சார்புநிலையை உணர்வுபூர்வமாக எதிர்க்கவும்
5. வலுவான மெய்நிகர் கூறுகளை வடிவமைக்கவும்
6. மாநாட்டை மாற்றுத்திறனாளிகள் அணுகும்படி செய்யவும்
7. உங்கள் சமூகத்தின் பன்மொழித்தன்மைக்கு இடமளிக்கவும்
8. உள்ளடக்கிய தகவல் தொடர்பு உத்தியை உருவாக்கவும்
9. உள்ளடக்கிய இலக்குகளை ஆதரிக்க போதுமான நிதி ஆதாரங்களை ஒதுக்கவும்
10. மாநாட்டை நீண்ட கால உள்ளடக்கச் செயல்முறையின் ஒரு பகுதியாக ஆக்கவும்

ฉบับภาษาไทย [Thai version]

กฎ 10 ข้อของการจัดงานสัมมนาที่ทุกคนสามารถมีส่วนร่วมได้อย่างเท่าเทียม

Kewalin Samart, Natnaree Yolnava, Wanprakai Kaewphrae, Napak Pacharadomrongsuk

เกวลิน สามารถ, ณัฏฐ์นรี ยลนาวา, วรรณประกาย แก้วแพร่, ณภัค พัชรดำรงสุข

งานสัมมนาเป็นพื้นที่สำหรับผู้คนในการปะทะสังสรรค์และแลกเปลี่ยนองค์ความรู้ทั่วไป
ตลอดจนความเชี่ยวชาญทั้งทางวิชาการและเทคนิคเฉพาะด้านต่างๆ
การเข้าร่วมงานสัมมนาจะช่วยเพิ่มโอกาสในการขยายเครือข่ายความร่วมมือของผู้คนจากสาขาต่างๆ
ซึ่งเป็นประโยชน์อย่างมากต่อการพัฒนาต่อยอดและสร้างสรรค์งานในระยะยาว
อย่างไรก็ตามในปัจจุบันโอกาสการเข้าถึงและเข้าร่วมงานสัมมนายังไม่เท่าเทียมสำหรับทุกคน
ในบทความนี้ผู้เขียนขอเสนอกฎ 10 ข้อ สำหรับการจัดงานสัมมนาที่ทำให้ทุกคนสามารถเข้าถึงและมีส่วนร่วมได้
เมื่อไม่นานมานี้ทางคณะผู้เขียนได้จัดงานสัมนาโปรแกรมภาษาอาร์สำหรับการคำนวณทางสถิติ (useR!) ประจำปี
พ.ศ. 2564 ถึงแม้งานสัมมนาจะถูกจัดขึ้นในช่วงวิกฤติโรคระบาดโควิด 19 (COVID-19)
เราได้พยายามอย่างสุดความสามารถในการจัดงานสัมมนาอย่างมีคุณภาพและสามารถดึงดูดผู้เข้าร่วมจากหลายภาคส่วนหลากหลายสาขาอาชีพ ทั้งภาคการศึกษา อุตสาหกรรม และองค์กรที่ไม่แสวงหากำไรจากหลากหลายทวีปทั่วโลก
ทางเราสนับสนุนและตั้งเป้าหมายอย่างชัดเจนในการสร้างงานสัมมนาให้เป็นพื้นที่ที่ทุกคนสามารถมีส่วนร่วมได้อย่างเท่าเทียม
จากประสบการณ์การจัดงานสัมมนาครั้งที่ผ่านมาเราได้เรียนรู้และนำเอาข้อผิดพลาดมาวิเคราะห์ร่วมกันเพื่อแสวงหาแนวทางแก้ไขอย่างเป็นรูปธรรม
ทั้งนี้เราได้รวบรวมแนวความคิดต่างๆไว้เป็นแบบแผนและกลยุทธ์นำร่องสำหรับการจัดงานสัมนาทั่วทุกมุมโลกที่ส่งเสริมความหลากหลายและการมีส่วนร่วมดังต่อไปนี้

กฎ

1. สร้างวิสัยทัศน์ของการส่งเสริมและยอมรับความแตกต่าง
2. สร้างบรรยากาศที่เป็นมิตรและให้การต้อนรับกับทุกคน
3. จัดตั้งฝ่ายเฉพาะเพื่อดูแลด้านการมีส่วนร่วมและความหลากหลาย
4. ตระหนักและร่วมกันต่อต้านอคติทางความคิดที่อาจเกิดขึ้นในงานสัมมนา
5. ออกแบบรูปแบบการสัมนาออนไลน์ที่มีประสิทธิภาพใช้งานง่าย
6. สร้างงานสัมมนาที่รองรับและอำนวยความสะดวกแก่ผู้พิการ
7. สร้างงานสัมมนาที่รองรับความหลากหลายทางภาษา
8. สร้างกลยุทธ์การสื่อสารที่รองรับการมีส่วนร่วมอย่างมีประสิทธิภาพ
9. จัดสรรค์งบประมาณที่เพียงพอเพื่อสนับสนุนเป้าหมายในการส่งเสริมความหลากหลายและการมีส่วนร่วม
10. สร้างงานสัมมนาให้เป็นส่วนหนึ่งของแผนการสนับสนุนการมีส่วนร่วมในระยะยาว

2. Rules proposed in the process of shaping these ten simple rules

Person 1

1. Embrace all dimensions of diversity
2. Have an inclusive organizing team
3. Go beyond your limited networks to find the best people for everything
4. Have registration rates according to the cost of living of participants
5. Do not give scholarships or grants to attend. Give fee waivers or discounts without tedious application forms.
6. Make the conference accessible to all
7. Put more effort in promoting your conference among groups of people who are usually underrepresented
8. Have an important online component of the conference
9. Don't let English be a barrier for good quality participation
10. Have a code of conduct team

Person 2

1. Diverse speakers, panelists, and conference coordinators, not just gender diversity but topic, ethnicity, race, physical appearance etc.
2. Cost of conference to be affordable in all countries promoting inclusive participation
3. A Code of Conduct response team and a strong Code of Conduct
4. Online / Virtual relay so that people from all over can attend
5. Timezone inclusive, so people from different time zones don't feel left out
6. Make all the materials available for free after the conference to people
7. This may be very simple but captions also helped me understand more
8. Hand holding for newbies as some might feel it is totally overwhelming
9. Help for people who cannot see colours / colour blind
10. Support for childcare or internet support
11. Communicating conference details effectively

Person 3

1. Embrace pluralism
2. Rethink hierarchies from inside
3. Recognize and fight bias
4. Democratize information and make labor collective
5. Challenge power during the process
6. Make the conference accessible
7. Make the conference inclusive
8. Include communities
9. Be committed to the community
10. Be accountable and permanent

Person 4

1. Define your accessibility standards
2. Be mindful of heterogeneous technical infrastructure
3. Provide accessibility guidelines
4. Encourage presenters to create accessible presentations
5. Be open to multilingual presentations
6. Provide captions of recorded and live talks
7. Ask for materials in advance and make them available before, during, and after the conference
8. Look for accessible chat platforms
9. Keep an accessible social media
10. Provide a contact media for accessibility questions

Person 5

1. The decision to have an inclusive conference needs to be shared and discussed in the organizing committee, from the beginning of the organization process
2. Have a diverse organizing team
3. Organizers contributing to diversity and accessibility committees also need to be “diverse”

4. Set your inclusion, diversity, and accessibility expectations for people external to the organizing committee

5. Have and enforce a Code of Conduct (CoC)

6. Accessibility

7. Offer fee waivers and financial grants, and different registration costs according to the income level

8. Connect with communities and local associations

9. Timezones and locations

10. Plan, balance and keep working to improve inclusion in the next edition of the conference!

Person 6

1. Define what diversity and inclusion mean for your conference

2. Create a safe and welcoming environment

3. Gather an inclusive and diverse organizing team

4. Unbias your spotlight roles

5. Actively reach out and promote the conference to people who have been systematically excluded

6. Make the conference accessible to all, from start to end

7. Have a strong online component of the conference

8. Expand the language possibilities for your conference

9. Allocate financial resources to support the presence of diverse people in every space of the conference

10. Aim for Progress, not Perfection

11. Don’t be afraid to break the rules

Person 7

1. Build an inclusive and diverse organizational team

2. Build a hybrid conference with the possibility for virtual attendance

3. Create specific fees for people with low incomes or disabilities

4. Create a diversity and inclusion team in the organizational team

5. Made it clear that people from all backgrounds and from different countries are welcomed

6. The conference should support multiple languages

Person 8

1. Define your vision and set bold but realistic targets
2. Elaborate a Diversity Statement
3. Build a diverse Team
4. Think about impediments to different participant groups, from abstract submission to attending the conference
5. Think and reach beyond the “usual suspects” when selecting the program committee, keynote speakers, award reviewers, session chairs
6. Have an online Component
7. Adopt a “Pay what you can” approach
8. Target underserved groups in your communication
9. Have a code of conduct and train a team to enforce it
10. Aim for progress, not perfection

Person 9

1. Prioritize accessibility throughout planning/organizing
2. Reserve decision-making roles for members of historically marginalized communities
3. Establish community norms early and that are agreed upon among conference organizers/volunteers by consensus
4. Make room for historically marginalized voices in planning/organizing meetings and other collaborative sessions to allow everyone to participate equally
5. Meet people where they are in their ability to contribute, and prepare to be nimble
6. Prioritize leadership, sponsorship, and attendance from around the world, particularly from the Global South
7. Reduce financial barriers by adjusting the cost of conference participation according to country and relative financial status, and by offering fee waivers/scholarships
8. Ask conference speakers and instructors to agree to accessibility standards for their materials and presentations, and provide guidelines to support them
9. Prioritize safety by establishing security protocols and Codes of Conduct both for planning/organizing and for conference attendance
10. Allow free and open access to content after the conference to participants that could not attend in real-time

Person 10

1. Ensure representation and diversity among the organizing committees, keynote speakers, presenters, session chairs, panelists, and participants
2. Set a budget that built-in support for diversity and inclusion
3. Plan for accessibility from the early stage in addition to accommodating requests
4. Develop a code of conduct or a harassment policy for the conference
5. Be public about your commitment to equity and inclusion
6. Use inclusive images and specific language
7. Celebrate and provide credit and visibility to volunteers/organizers
8. Outreach/promotion plan that reaches communities that are traditionally excluded in scientific meetings
9. Meeting the need of a linguistically diverse audience
10. Assess whether equity and inclusion goals were met during the meeting

Person 11

1. Have a diverse organizing committee
2. Use feedback from previous conferences or other conferences in your field to identify barriers to inclusion and plan around them
3. Keep attendance costs low
4. Make information about the Code of Conduct prominent starting early in the abstract submission process
5. Offer content in languages other than English
6. Live caption all events
7. Be careful how you talk about minoritized people
8. Explain the accessibility practices on the website
9. Have a contact email for questions about accessibility
10. Have a screen-reader accessible template for slides
11. Assertively set tone of inclusion
